# Study on Adsorption of Cu and Ba from Aqueous Solutions Using Nanoparticles of *Origanum* (*OR*) and *Lavandula* (*LV*)

**DOI:** 10.1155/2018/3936178

**Published:** 2018-09-09

**Authors:** Ghadah M. Al-Senani, Foziah F. Al-Fawzan

**Affiliations:** Department of Chemistry, College of Science, Princess Nourah Bint Abdulrahman University, Riyadh, Saudi Arabia

## Abstract

Wild herbs (*Origanum* (*OR*) and *Lavandula* (*LV*)) were used as environment-friendly adsorbents in this study. The adsorbents were used for adsorption of Cu and Ba from water. The adsorption of heavy metals onto *OR* and *LV* was dependent on particle size, dose, and solution pH. The diameter of adsorbent particles was less than 282.8 nm. The adsorption follows second-order kinetics. Langmuir and Freundlich models have been applied to describe the equilibrium data, and the thermodynamic parameters, the Gibbs free energy, ∆*G*°, enthalpy, ∆*H*°, and entropy, ∆*S*°, have been determined. The positive value of ∆*H*° suggests that the adsorption of heavy metals by the wild herbs is endothermic. The negative values of ∆*G*° at all the studied temperatures indicate that the adsorption is a spontaneous process. It can be concluded that *OR* and *LV* are promising adsorbents for the removal of heavy metals from aqueous solutions over a range of concentrations.

## 1. Introduction

The adsorption of toxic substances resulting from the accumulation of industrial wastes is important and one of the most dangerous challenges facing the environment and society nowadays. One of the most significant contaminants affecting water resources is heavy metals. These metal ions present a significant risk to animals and humans because of their high toxicity at both low and high concentrations in soil and water. The search for new techniques to remove these contaminants has involved both chemical and biological methods.

Contaminating materials have become increasingly dangerous with increasing technological development, and need for diverse heavy metals, for example, in ore processing and other modern industries, prompted the organizations concerned with the preservation of the environment to develop restrictions and laws for laboratories and concerning the treatment industrial waste before its release into the environment so that toxic materials do not exceed the allowable limits. Researchers have, thus, devoted efforts to find effective methods for the removal of contaminants from waste, but these processes are often economically expensive; therefore, we must find ways to bypass conventional and high-cost advanced adsorption technologies.

The adsorption of contaminants on the surfaces of solids is an effective method for heavy metal remediation. Activated carbon is considered to be an efficient, competitive material for this task. However, the cost of production is still high; thus, many researchers have begun to search for alternative adsorbents made from local natural materials. Plants are one type of alternative material that can be used to remove heavy metal ions from water systems and soil.

The current proliferation of technology and development of science has been an enormous boon for humans. They have become dependent on technology and scientific development in various aspects such as daily activities, trade, industry, and work. Heavy metals are structural elements such as lead, zinc, arsenic, cadmium, copper, titanium, cobalt, lithium, aluminum, and mercury and can be in the form of metals or dissolved salts.

These metals are present in the environment in air, water, and soil. For example, factory chimneys release metal oxides into the air, thus transmitting heavy metal pollution to humans, animals, and plants. In addition, car exhausts release lead oxides, resulting from the combustion of tetraethyl lead, into the atmosphere, and this is one of the most widespread routes to leading contamination of marine organisms with metals and the transit of these contaminants via sea fishing to humans and animals. Furthermore, agricultural soil is one of the most important sources of food polluted by heavy metals, which arises through the irrigation of crops with polluted water or the use of pesticides. In this situation, the metals are transmitted through the vascular system of plants and fruits. Therefore, field crops irrigated with drainage water polluted by heavy metals are one of the most important and most dangerous sources for the entry of toxic heavy metals into the human body [1].

There are many methods for adsorption of heavy metals from the environment, both chemical and physical. However, some of these are not economically feasible. Therefore, it is necessary to investigate low-cost, effective alternatives. The adsorption of heavy metals by the adsorption technology is a good alternative, and it is used in the treatment of wastewater and soil. To compare the adsorbent substances, the cost, as well as effectiveness, must be considered. Activated carbon is a highly effective alternative used for the adsorption of heavy metals from wastewater, but it is soluble under extremely acidic conditions [2].

Consequently, there is an increased interest from researchers into low-cost, effective alternative adsorbents. Many natural materials used in the adsorption of heavy metals (cadmium, copper, chromium, lead, nickel, cobalt, and lithium), such as *Diplotaxis harra*, *Glebionis coronaria*, coffee grounds, banana peel, fruit and vegetable peels, cactus, rice straw, wheat straw, and salvinia plant, have been discovered [3–8].

The aim of this work is to study the adsorption of Cu and Ba from water using microparticles of *Origanum* (*OR*) and *Lavandula* (*LV*), which are considered environmentally safe and low-cost. In this study, we use thermodynamic, kinetic, equilibrium, and adsorption isotherms in the analysis of the adsorption behavior.

## 2. Experimental

### 2.1. Materials

All the chemicals used in this study were of analytical grade. Cu(Cl)_2_·4H_2_O (98%) and Ba(Cl_2_)·4H_2_O (90%) were purchased from Sigma-Aldrich (Germany).

### 2.2. Preparation of the Adsorbents

**Figure 1:**
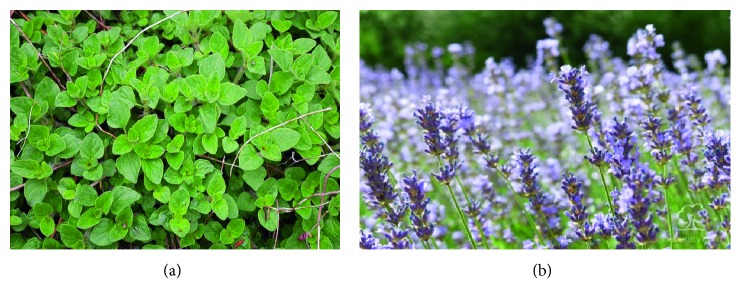
(a) *Origanum* (*OR*) and (b) *Lavandula* (*LV*).

*Origanum* (Figure 1) is a leafy plant used as a kind of aromatic spices and flavorings in foods, as well as the benefits of medical oregano. It also contains a variety of antioxidant compounds, such as rosmarinic acid. The chemicals in oregano are those that give it distinctive aroma and flavor. It includes thymol, albinin, limonene, carvacrol, and carophyllene [9].

*Lavandula* (*LV*Figure 1) is a plant that grows and prefers sunny open areas. Lavender is used for insomnia, nervousness, and depression [10].

The wild herbs used in this study were purchased from a perfumer. The dried herbs were then cut into small pieces and milled to a powder using a laboratory planetary ball mill (DECO-PBM-V-0.4 L). The powders were sieved into particles of 50, 100, 200, 300, and 400 *μ*m using an Octagon D200 digital sieve shaker. The adsorbents were stored in glass bottles for further use without any pretreatment.

### 2.3. Methods

For the adsorption study, distilled water was used to prepare various solutions at the desired concentrations from the stock solution. The adsorption experiments were performed in a series of flasks containing 100 mL solutions of metal ions at desired concentration and mass of adsorbent herbs. The mixtures were shaken for 16 h at 120 rpm using a shaker (“Rotaterm” orbital and linear shaker). The mixtures were filtered, and the heavy metals concentrations were determined by inductively coupled plasma (ICP) mass spectrometry. The effect of adsorbent doses from 0.1 to 2 g on the metal adsorption was studied. The initial concentration was 10 mg/L. The effect of pH on the heavy metal adsorption process was investigated at pH values from 3 to 12, adjusted either with 1 M·HCl or 1 M·NaOH using a pH meter, to monitor the change. In addition, the effect of the adsorbent particle size was investigated from 400 to less than 50 *μ*m, and the contact time was varied between 1 and 3 h. The effect of temperature was studied from 25° to 60°C for 3 h using 0.5 g of the adsorbent at an initial metal concentration of 10 mg/L.

The adsorption equilibrium was investigated for different metal concentrations between 1 and 100 mg/L. In addition, kinetic experiments were conducted with 10 mg/L metal and 0.5 g of the adsorbent at room temperature with stirring at 120 rpm for 6 h.

The adsorption at equilibrium, *q*_e_ (mg/g), was calculated by using the following equation:

(1) $$\begin{array}{c} q_{\text{e}}=\frac{\left(C_{0}-C_{\text{e}}\right)V}{m}, \end{array}$$

where *C*_0_ and *C*_e_ (mg/L) are the liquid-phase heavy metal concentrations initially and at equilibrium, respectively; *V* is the volume of the solution (L); and *m* is the mass of the dry adsorbent (g).

The percentage of metal adsorption (Ads_HM_%) from the solution was calculated as follows:

(2) $$\begin{array}{c} {\text{Ads}}_{\text{HM}}\%=\frac{\left(C_{0}-C_{\text{e}}\right)}{C_{0}}\times 100. \end{array}$$

The pseudo-first-order and pseudo-second-order adsorption kinetic models were applied to describe the behavior of the adsorbent.

The equilibrium data were then fitted using the Langmuir and Freundlich isotherm models.

The thermodynamic parameters calculated to describe the adsorption process onto *OR* and *LV* include the changes in standard enthalpy (∆*H*°), standard entropy (∆*S*°), and standard free energy (∆*G*°).

### 2.4. Characterization of the Adsorbents

Fourier-transform (FT-IR) analysis was used to determine the functional groups in the adsorbents. The particle size of adsorbents was measured using an XRD. The initial metal concentrations of the adsorbents were determined for Cu and Ba from ICP measurements.

## 3. Results and Discussion

### 3.1. Characterization of the Adsorbents

**Figure 2:**
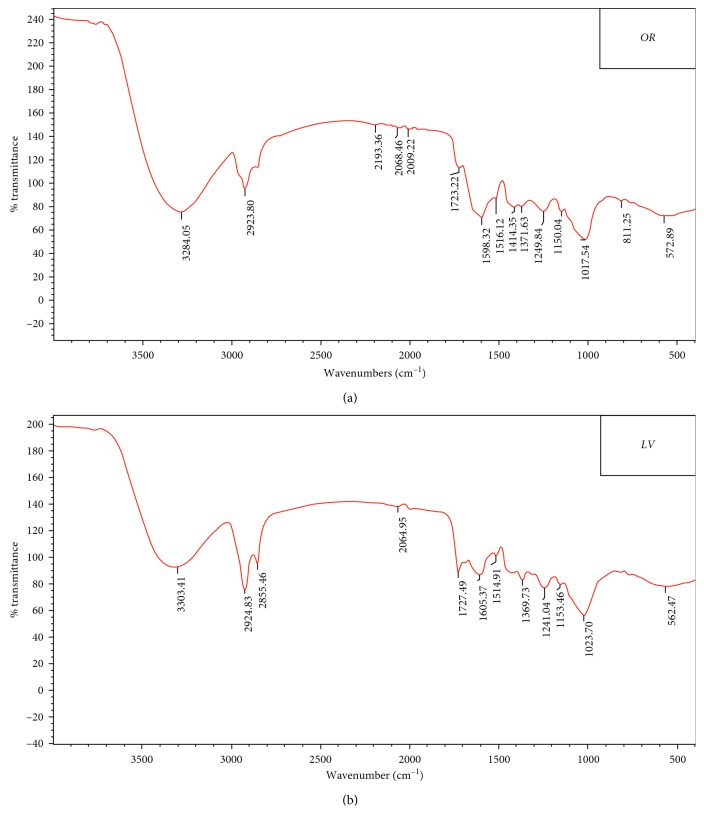
FT-IR spectra of the functional groups in (a) *Equisetum*, *OR*, and (b) *Teucrium*, *LV*.

Figure 2 shows the FT-IR absorption bands of the adsorbent. The peaks indicate the presence of functional groups containing O-H and N-H at 3300 cm^−1^ wavelength and carboxylic acid (−COOH) groups at 1600 cm^−1^ and 1150 cm^−1^ wavelengths, which play a major role in the adsorption process and efficiently adsorb metal ions via ion exchange [11].

**Figure 3:**
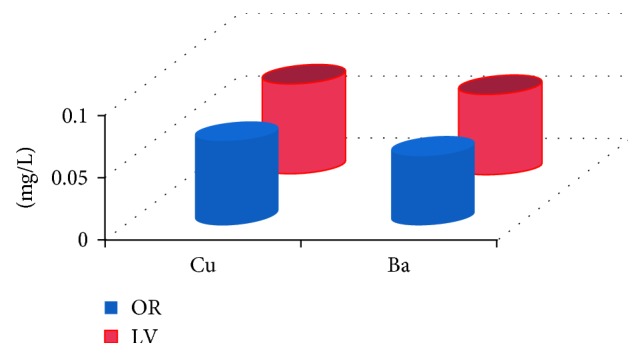
Concentration of heavy metals in the adsorbent.

The mineral composition of biomass ash commonly shows a lowest amount of Cu and Ba (not exceeding 0.072 mg/L) in *OR*, as does *LV*, and hence the Cu and Ba were selected for further study (Figure 3).

**Figure 4:**
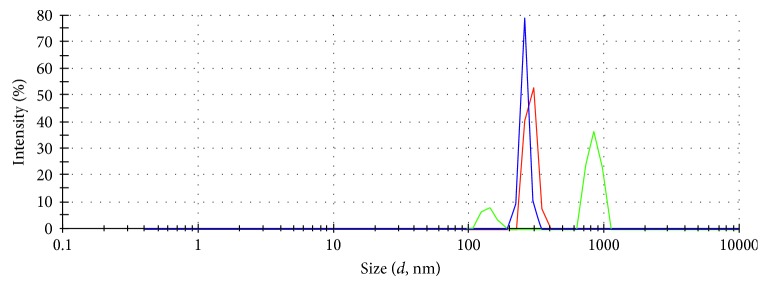
XRD of the particle size of adsorbents.

The smallest size obtained after milling the wild herbs was 50 *μ*m.  Figure 4 shows the size of adsorbent particles that had been ground to less than 50 *μ*m. The diameter of the particle is less than 282.8 nm, which is smaller than the size reached by milling, and smaller particles can increase the effectiveness of adsorption.

The small particle size was characterized by fine porosity and a large internal surface area, while the powder form has larger diameter pores and smaller internal surface area. A smaller pore size results in stronger and greater adsorption capacity because the small adsorbent particle size reduces the pathway for both mass transport and internal diffusion of the adsorbent inside the adsorbents [12].

### 3.2. Effect of Particle Sizes of Adsorbent

**Figure 5:**
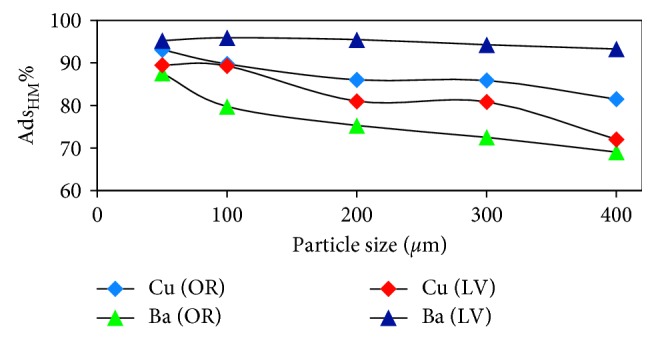
The effect of particle size on the metal ion adsorption by *OR* and *LV* (initial pH, 120 rpm, 10 mg/L of metals, 0.5 g of adsorbents, and 25°C for 16 h).

The experimental effect of particles on metal adsorption was investigated for five sizes (400 to less than 50 *μ*m) at room temperature and initial pH (5 and 7 for Cu and Ba, respectively).  Figure 5 indicates that metal adsorption increased with decreasing particle size for *OR* and *LV*. This behavior can be attributed to the increased internal surface area with decreasing particle size [12].

This behavior arises because most of the internal surface of these particles can be used for adsorption. The smaller particle size gives higher adsorption rates because metal ions have a short path for transfer inside the pores of the small particle adsorbents [13].

However, it is expected that the use of the smaller particle size should give greater removal rate because of the increased surface area, and as the particle size increases, the number of small pores increases. The particle dimensions determine the propagation distance, because the dimensions of the adsorbent particles obtained by standard sieves depend on the particle length and width; thus, this explains the decreased adsorption of metals with increasing adsorbent particle size [14].

### 3.3. Effect of Adsorbent Dosage

**Figure 6:**
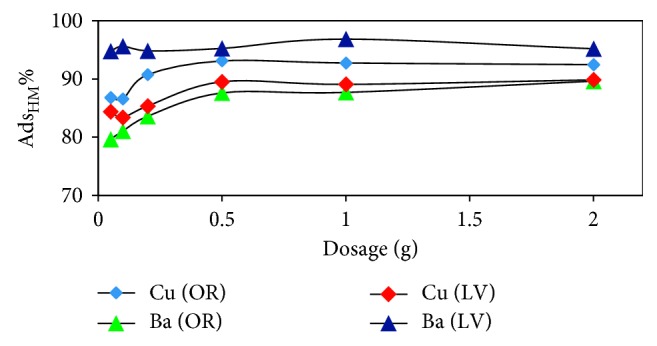
The effect of dosage on the metal ion adsorption by *OR* and *LV* (initial pH, 120 rpm, 10 mg/L of metals, 50 *μ*m size of adsorbents, and 25°C for 16 h).

The effect of the adsorbent doses on heavy metal adsorption by *OR* and *LV* was studied. As shown in Figure 6, the adsorption percentage of Cu and Ba increased with increase of adsorbent doses. The increase in adsorption percentage with increasing adsorbent dose arises because of the increased availability of exchange sites or surface areas at higher concentrations of adsorbents [15]. Similar behavior has been observed in previous studies and arises from the effect of interactions between mineral ions and adsorbent. The increased dose is consistent with the greater area and larger number of adsorption sites [16].

### 3.4. Effect of pH

**Figure 7:**
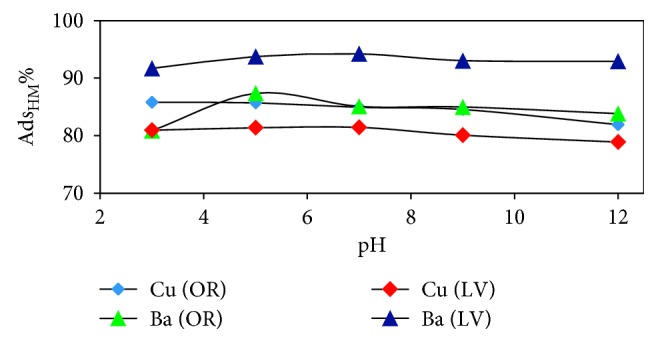
The effect of pH on the metal ion adsorption by *OR* and *LV* (120 rpm, 10 mg/L of metals, 50 *μ*m size and 0.5 g of adsorbents, and 25°C for 16 h).

As shown in Figure 7, the percent adsorption of Cu and Ba increased significantly with increasing pH from 3 to 5, and then stabilized until reaching pH 7; after that, the adsorption was a slight decrease above pH 7 for both adsorbents *OR* and *LV* [15].

## 4. Adsorption Kinetic Model

To understand the rate and type of adsorption on adsorbents, we studied several kinetic models.

### 4.1. Pseudo-First-Order Kinetic Model

The pseudo-first-order model is expressed by the following equation:

(3) $$\begin{array}{c} \log\left(q_{\text{e}}-q_{t}\right)=\log\;q_{\text{e}}-\frac{K_{1}t}{2.303}, \end{array}$$

where *q*_e_ and *q*_*t*_ represent the amount of metal ions adsorbed at equilibrium and at time, *t*, respectively and *K*_1_ is pseudo-first-order rate constant.

**Figure 8:**
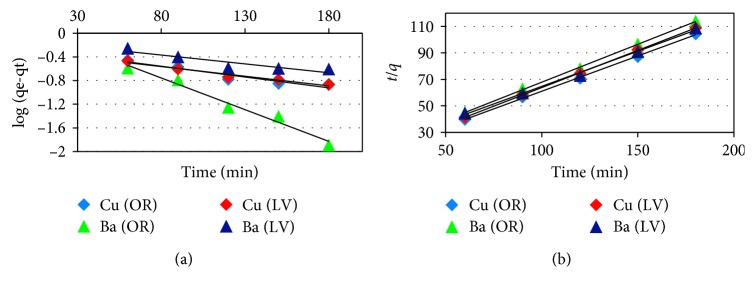
The plot of (a) pseudo-first order (POF) and (b) pseudo-second order (POS) for the metal ion adsorption onto *OR* and *LV* (initial pH, 120 rpm, 10 mg/L of metals, 50 *μ*m size and 0.5 g of adsorbents, and 25°C at time 60, 90, 120, 150, and 180 min).

As shown in Figure 8 and Table 1, the regression coefficient (*R*^2^ ≤ 0.96) indicates that the experimental data accurately support the first-order model to describe the adsorption kinetics of Cu ions, but do not support it with Ba ions. However, we found that the typical values of *q*_m_ are lower than the experimental values for Cu ions. This indicates that both metal ions and the adsorbent are involved in the adsorption process [17]. Therefore, the first-order model may not be suitable for explaining the adsorption kinetics of ions on the adsorbents. Similar results were obtained for the kinetics of adsorption of different metal ions on other adsorbents [17–19].

### 4.2. Pseudo-Second-Order Kinetic Model

The pseudo-second-order model is expressed by the following equation:

(4) $$\begin{array}{c} \frac{t}{q_{t}}=\frac{1}{K_{2}{q_{\text{e}}}^{2}}+\frac{t}{q_{\text{e}}}, \end{array}$$

where *K*^2^ is the pseudo-second-order rate constant.

The experimental data showed the best fit with the highest correlation coefficients (*R*^2^ ≥ 0.996) for the pseudo-second-order model for each of the metal ions. In addition, the experimental values were well matched with the calculated data (Table 1). Therefore, the experimental data accurately support the pseudo-second-order kinetic model for the adsorption of metal ions.

This finding indicates that the rate-limiting step was not the resistance of the boundary layer [8]. Thus, the rate-limiting step is chemisorption involving valence forces through the exchange of electrons between the metal ions and the different functional groups in the adsorbent [20]. This is consistent with the observed rapid adsorption.

As shown in Figure 8, the kinetic analysis for the adsorption of metal ions on the catalysts revealed one step: the rate of adsorption in the equilibrium state. This phenomenon indicates that the chemical adsorption is the dominant step on the adsorption process [11, 21]. The adsorption half-time (*t*1/2) for the adsorption of half the metal ions in minutes was between 13.45 and 21.80 min, indicating significant affinity of the adsorbent molecules for the adsorbate [8].

## 5. Adsorption Isotherm

**Figure 9:**
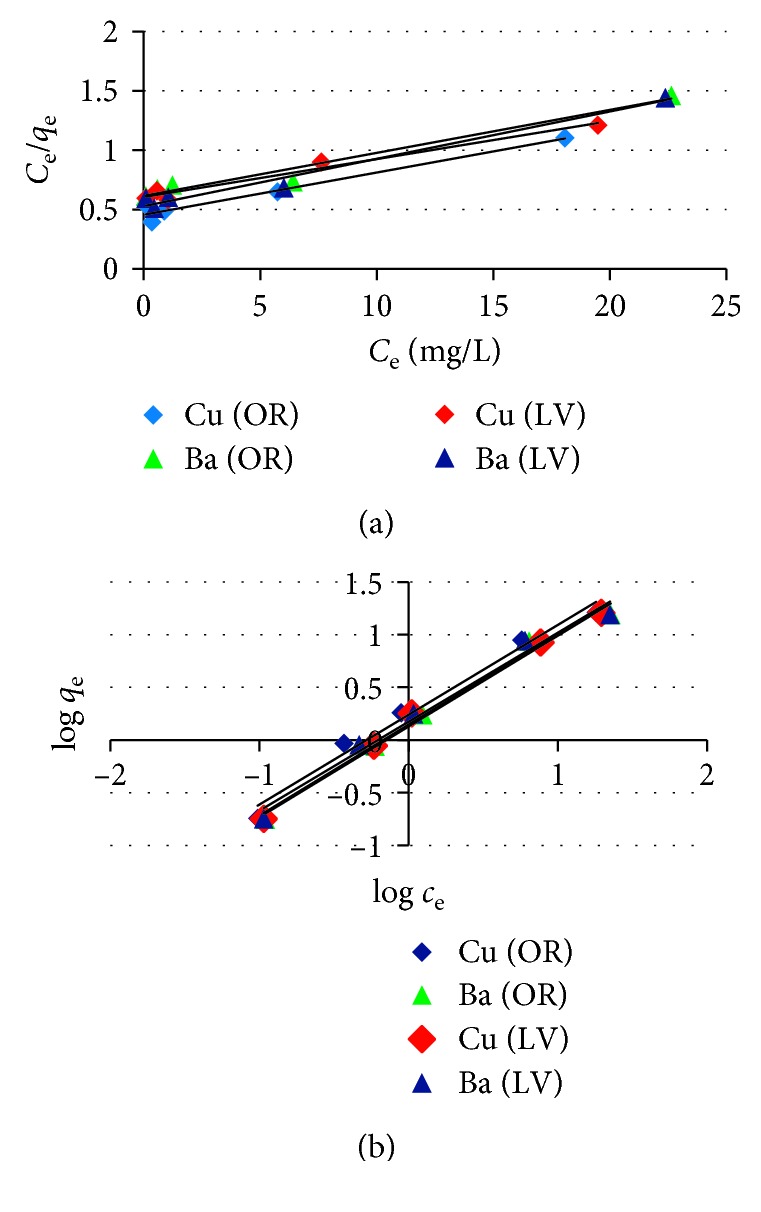
The (a) Langmuir and (b) Freundlich isotherms for the metal ion adsorption onto *OR* and *LV* (initial pH, 120 rpm, 50 *μ*m size of adsorbents, and 25°C for 16 h).

To understand the mechanism of metal ion adsorption from an aqueous solution by the adsorbents, we tested several adsorption isotherm models. The Langmuir adsorption isotherm was applied in accordance with the following equation:

(5) $$\begin{array}{c} \frac{C_{\text{e}}}{q_{\text{e}}}=\frac{1}{K_{\text{L}}q_{\text{m}}}+\frac{C_{\text{e}}}{q_{\text{m}}}, \end{array}$$

where *C*_e_ is the adsorbate equilibrium concentration, *q*_e_ is the observed adsorption capacity at equilibrium, *q*_m_ is the maximum adsorption capacity, and *K*_L_ is the adsorption equilibrium constant. The Langmuir adsorption isotherm showed a linear relationship between *C*_e_/*q*_e_ and *C*_e_ for the adsorption of metals on *OR* and *LV* (Figure 9), and the slope and *R*^2^ data are presented in Table 2. The plot exhibited good correlation coefficient and linearity. The *R*^2^ values are very close to unity, indicating strong correlation with the Langmuir adsorption isotherm. The maximum adsorption capacity of the adsorbent, *q*_m_ (mg/g), for metal ions is high, which can be explained by their noncovalent interaction with ions. Another reason may be the presence of carboxylic acid-containing sugars in the herbs, which bind the metal ions [22]. So, the values of *R*_L_ were calculated from the following equation:

(6) $$\begin{array}{c} R_{\text{L}}=\frac{1}{1+K_{\text{L}}q_{\text{m}}}\log\;C_{\text{e}}. \end{array}$$

*R*_L_ ≤ 1, indicating that the Langmuir isotherm is appropriate for the adsorption process. For a single adsorption system, *C*_0_ is usually the highest concentration of the liquid phase encountered [23].

The Freundlich adsorption isotherm is given by the following equation:

(7) $$\begin{array}{c} \log\;q_{\text{e}}=\log\;K_{\text{F}}+\frac{1}{n}\log\;C_{\text{e}}, \end{array}$$

where *n* is the Freundlich equilibrium coefficient; values of log *q*_e_ are plotted against log *C*_e_ (Figure 9). The slope and *R*^2^ data are presented in Table 2. In the slope of the Freundlich plot, 1/*n* is greater than 1, which indicates that adsorption is cooperative. Higher values of *K*_F_ and *n* indicate a higher affinity for the adsorption of metals [4]. Values of 1/*n* < 1 indicate the favorable adsorption of all metals, and values of *n* within the range 1–10 represent good adsorption and a favorable physical process. Higher values of *K*_F_ indicate a high adsorption intensity [24].

From the Langmuir and Freundlich isotherms, it is clear the metal ions were strongly adsorbed in the pores of the herbs.

## 6. Thermodynamic Parameters

In order to understand the nature of adsorption, the thermodynamic parameters such as free energy change (∆*G*°), enthalpy change (∆*H*°), and entropy change (∆*S*°) could be calculated using the following equations:

(8) $$\begin{array}{c} \Delta G{}^{\circ}=-\text{RT}\ln\;\;K_{\text{eq}},\\ \ln\;\;K_{\text{eq}}=\frac{\Delta S{}^{\circ}}{R}-\frac{\Delta H{}^{\circ}}{\text{RT}},\\ \Delta G{}^{\circ}=\Delta H{}^{\circ}-T\Delta S{}^{\circ}. \end{array}$$

**Figure 10:**
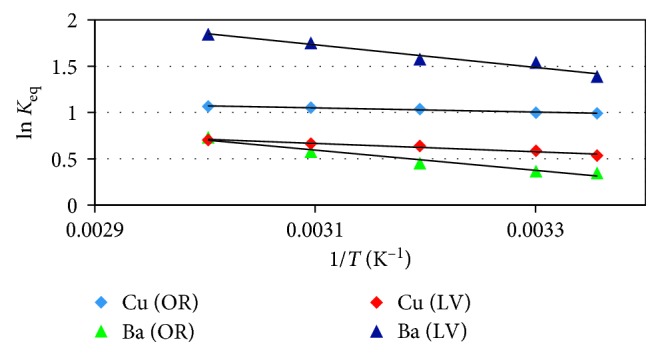
van 't Hoff plots for the metal ion adsorption onto *OR* and *LV* (initial pH, 120 rpm, 10 mg/L of metals, and 50 *μ*m size and 0.5 g of adsorbents for 16 h).

The values of the thermodynamic parameters are shown in Table 3. The linearized van 't Hoff plots of ln *K*_eq_ versus 1/*T* are shown in Figure 10.

The values of ∆*H*° and ∆*S*° were determined from the slope and intercept of the linear plot.

The parameters indicate that the adsorption process is spontaneous and endothermic. The positive value of ∆*S*° indicates an increase in the degrees of freedom (or disorder) of the adsorbed species for Cu and Ba ions.

The negative values of ∆*G*° indicate that the thermodynamic process was spontaneous and feasible for Cu and Ba ions [8]. Moreover, the increase in negative ∆*G*° values with increasing temperature shows an increased probability of adsorption at higher temperatures, which is consistent with an earlier report [25].

## 7. Conclusion

In this study, the adsorption of Cu and Ba onto *OR* and *LV* was investigated with respect to the particle size, dosage of the adsorbent, and pH. Characterization of the adsorbents revealed the presence of carboxylic groups that were involved in the adsorption of the metal ions. Adsorption was maximum with 50 *μ*m particle size and 0.5 g of the adsorbent. Metal ion adsorption onto wild herbs follows the pseudo-first-order and pseudo-second-order kinetic models. The adsorption isotherm data for the Cu and Ba ions fitted well with the Langmuir and Freundlich models. The thermodynamic parameters show that the adsorption process was feasible, spontaneous, and endothermic in nature for Cu and Ba. Hence, the wild herbs can be used as a low-cost adsorbent for the removal of heavy metal ions from aqueous solutions. Further study is warranted to evaluate the potential of the adsorbents for heavy metal removal from aqueous solutions.

## Figures and Tables

**Table 1:** Kinetic model parameters for the metal ion adsorption onto *OR* and *LV*.

Kinetic model	Parameter	Adsorbent (*OR*)		Adsorbent (*LV*)	
		Cu	Ba	Cu	Ba
	*q*_e,exp_ (mg/g)	1.86	1.75	1.79	1.90

Pseudo-first order	*q*_e,calc_ (mg/g)	0.54	0.52	0.50	0.74
	*K*_1_ × 10^3^(min^−1^)	8.52	13.36	7.60	6.91
	*R* ^2^	0.904	0.669	0.962	0.823

Pseudo-second order	*q*_e,calc_ (mg/g)	1.88	1.75	1.78	1.88
	*K*_2_ × 10^3^ (g/mg·min)	36.96	30.92	41.81	24.37
	h (mg/g·min)	0.13	0.09	0.13	0.09
	*t*1/2 (min)	14.43	18.52	13.45	21.80
	*R* ^2^	0.999	0.999	0.999	0.996

**Table 2:** Adsorption isotherm parameters for the metal ion adsorption onto *OR* and *LV*.

Adsorption isotherm	Parameter	Adsorbent (*OR*)		Adsorbent (*LV*)	
		Cu	Ba	Cu	Ba
Langmuir	*q*_m_ (mg/g)	28.17	27.62	31.25	25.06
	*K*_L_ × 10^3^ (L/mg)	77.8	58.80	52.84	75.53
	*R*_L_	0.56	0.63	0.65	0.57
	*R* ^2^	0.963	0.965	0.975	0.976

Freundlich	*K*_F_ (mg/g) (L/mg)^1/*n*^	1.74	1.35	1.40	1.50
	*n*	1.17	1.16	1.16	1.18
	*R* ^2^	0.983	0.988	0.994	0.982

**Table 3:** Thermodynamic parameters for the metal ion adsorption onto *OR* and *LV*.

Parameter	Adsorbent (*OR*)		Adsorbent (*LV*)	
	Cu	Ba	Cu	Ba
∆*H*° (J/mol)	1867.07	9096.35	3748.28	10161.4
∆*S*° (J/mol K)	14.52	33.14	17.16	45.89
∆*G*° (kJ/mol) at 298K	–2.46	–0.78	–1.36	–3.51
∆*G*° (kJ/mol) at 333K	–2.97	–1.94	–1.96	–5.12
*R* ^2^	0.966	0.964	0.968	0.957

## Data Availability

The data used to support the findings of this study are available from the corresponding author upon request.
